# Pediatric Emergency Department-based Food Insecurity Screening During the COVID-19 Pandemic

**DOI:** 10.5811/westjem.19488

**Published:** 2024-11-21

**Authors:** Stephanie Ruest, Lana Nguyen, Celeste Corcoran, Susan Duffy

**Affiliations:** * Hasbro Children’s Hospital, Division of Pediatric Emergency Medicine, Warren Alpert Brown University School of Medicine, Providence, Rhode Island; † Brown University, Providence, Rhode Island; ‡ Hasbro Children’s Hospital, Department of Pediatrics, Providence, Rhode Island

## Abstract

**Introduction:**

The emergency department (ED) is a safety net, caring for families who lack adequate access to food and other basic needs. The COVID-19 pandemic caused a dramatic rise in food insecurity (FI) nationally; however, little is known about the prevalence of FI among families seen in pediatric EDs (PED). In this study we aimed to determine the prevalence of FI, as well as awareness and utilization of supplemental food services, among families seen in an urban PED during the COVID-19 pandemic using an electronic screening survey.

**Methods:**

This was a cross-sectional survey of families screened for FI in an urban PED. An electronic survey was advertised to all families via posters placed in patient rooms and other locations in the PED between February–October 2022. Surveys in English and Spanish were accessed on personal electronic devices via QR codes. Six validated US Department of Agriculture household food security questions and sociodemographic questions were included. We calculated respondents’ food security and performed descriptive and bivariate analyses of patient sociodemographics and responses to FI questions.

**Results:**

Of 42,697 PED visits, 612 surveys were completed and analyzed (1.4%). Nearly 50% of respondents identified as White and non-Hispanic, with approximately 80% female. Thirty percent had a household income of <$25,000 and 32% between $25,000–<50,000. Among survey respondents, 56.7% demonstrated FI: 25% with low food security, and 31.7% with very low food security. We identified statistically significant differences in awareness and use of supplemental food services by FI status, household income, and primary language spoken.

**Conclusions:**

Nearly 60% of survey participants in an urban pediatric ED during the COVID-19 pandemic experienced food insecurity, substantially higher than previous reports. These results support the ED’s contributory role in FI screening, particularly during times of a public health crisis, and highlights the need for targeted outreach in this setting.

Population Health Research Capsule What do we already know about this issue?

*The COVID-19 pandemic caused dramatic increases in food insecurity (FI), affecting 1 in 4 families nationally.*

What was the research question?

*What was the prevalence of FI and use of supplemental food programs among families seen in a pediatric ED during the pandemic?*

What was the major finding of the study?

*56.7% of respondents had FI; 25% had low food security (95% CI 21.6–28.4) and 31.7% had very low food security (95% CI 28.0–35.6).*

How does this improve population health?

*Given the high prevalence of FI in this population, EDs have become important locations for FI screening.*

## INTRODUCTION

Food insecurity (FI) is a significant public health issue, dramatically worsened by the COVID-19 pandemic.^1^ ^,^ ^2^ Pre-COVID-19, an estimated 1 in 7 children in the US lived in households with FI. This strikingly increased to nearly *1 in 4* households with children during the early COVID-19 pandemic,^1^ ^,^ ^2^ with approximately 6.4 million children living in FI households in 2022.^2^ A survey performed one month into the COVID-19 lockdowns demonstrated FI among more than 40% of households of mothers with children ≤12 years of age.^3^ Although children may be shielded from directly experiencing FI by their caregivers,^2^ ^,^ ^4^ a nationwide survey of mothers with young children identified that in almost 20% of households with children <12 years of age, the children directly experienced FI.^3^

Given the myriad of physical and mental health implications of FI among children,^5^ the American Academy of Pediatrics (AAP) Promoting Food Security for All Children policy statement advises that pediatricians “play a central role” in both screening and advocacy for FI among their patients.^5^ The ED serves as a safety net for families experiencing poverty and barriers to resource access and utilization and who are at highest risk of FI and inadequately meeting basic needs. It has previously been reported that children with FI have higher odds of visiting an ED than those who do not.^6^ ^,^ ^7^ Thus, pediatricians and other clinicians seeing these at-risk patients in the ED have a critical opportunity to provide the recommended screening. Previous pre-COVID-19 studies have demonstrated the feasibility of pediatric emergency department (PED)-based FI screening,^7^ ^–^ ^10^ with a caregiver preference for electronic-based screening tools.^7^ ^,^ ^11^

Although studies in the general population have demonstrated marked increases in FI during the COVID-19 pandemic, the prevalence of FI among families in PEDs is not well understood. Thus, we aimed to describe the prevalence of FI among families in an urban PED during the COVID-19 pandemic with an electronic FI screening program using validated US Department of Agriculture (USDA) FI screening questions, and to describe the associated sociodemographics and caregiver awareness and use of supplemental food resources.

## METHODS

### Study Population and Design

This survey was performed in the Hasbro Children’s Hospital PED, the only children’s hospital, PED, and Level 1 trauma center in the state of Rhode Island. The institutional PED cares for over 55,000 children annually from Rhode Island as well as neighboring areas of Massachusetts and Connecticut, serving as a safety net for a region comprising a broad spectrum of rural, suburban, and urban communities. Approximately 48% of patients seen in the institutional PED annually are female, 47% identify as White, 14% Black, and nearly 40% Hispanic. Over 55% of patients have government insurance. Additionally, approximately 83% of patients report English as the preferred language with 15% reporting Spanish.

Recruitment posters with survey QR codes, displayed in both patient rooms and public waiting areas within the PED, invited parents and caregivers >18 years of age to answer questions about their family demographics and access to food via an electronic survey (Qualtrics LLC, Provo, UT). Recruitment posters were not available in rooms dedicated to psychiatric evaluations and critical care, or in rooms used for after-hours urgent care overflow patients seen by PED clinicians in non-PED locations. To accommodate potential language challenges, posters were written in English and Spanish with basic instructions to scan the QR code with a smartphone. Surveys were displayed between February–October 2022 based on the grant funding period and availability of trained study staff. No direct assistance was provided during the enrollment process. Responses were collected anonymously. All respondents were given the option of providing contact information to be eligible for a small incentive, a grocery gift card. All respondents, regardless of food security status, were also given the option of providing their contact information to be contacted by a hospital resource advocate after the ED visit for assistance with enrollment in food resource programs.

The survey included sociodemographic questions pertaining to the respondent and the child currently being seen in the PED (age, sex, race, ethnicity, primary language, household size and income, employment status, education, housing, respondent’s relationship to the child in the ED, insurance status, and the child’s access to pediatric primary care), and six questions from a validated USDA Household Food Security Survey Module (Figure).^12^ Respondents were also asked if they had ever “heard of” and/or were currently using supplemental food services (Women, Infants, & Children Program [WIC], Supplemental Nutrition Assistance Program [SNAP], local food banks, and free school-lunch programs), hereafter referred to as “awareness” and “utilization.”

**Figure.**
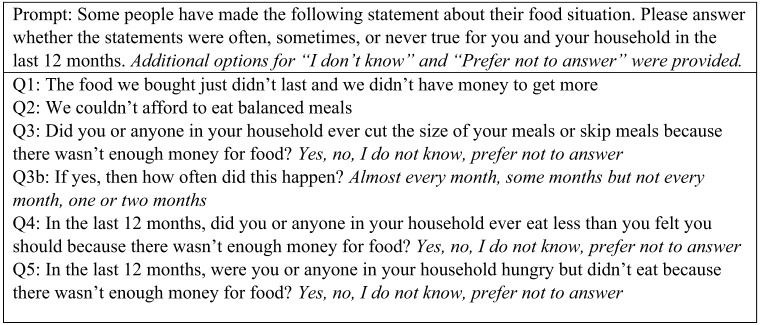
United States Department of Agriculture food insecurity screening questions.^a^ ^a^Food insecurity raw score is calculated as follows: Answers of “often” or “sometimes” for questions 1 and 2, “yes” on questions 3, 4, and 5, and “almost every month” or “some months but not every month” on Q3b are all coded as affirmative (yes). The sum of the affirmative answers to these 6 questions are used to calculate a raw score. Score of 0–1 denotes high or marginal food security, 2–4 denotes low food security, and 5–6 denotes very low food security.

We assigned each of the six USDA screening questions an individual score based on the responses, and we then calculated a FI “raw score” by the sum of the individual question scores for each of the FI screening questions, according to the USDA scoring guide.^12^ We calculated the FI raw score as follows: answers of “often” or “sometimes” for questions 1 and 2, “yes” on questions 3, 4, and 5, and “almost every month” or “some months but not every month” on Q3b are all coded as affirmative (yes). The sum of the affirmative answers to these six questions was then used to calculate the raw score. Score of 0–1 denotes high or marginal food security, 2–4 denotes low food security, and 5–6 denotes very low food security. Low food security indicates that the household “obtained enough food to avoid substantially disrupting their eating patterns or reducing food intake by using a variety of coping strategies, such as eating less varied diets, participating in federal food assistance programs, or getting food from community food pantries.”^2^ Very low food security indicates that household members reduced their food intake because of inadequate money or resources for food.^2^ ^,^ ^4^

We translated respondents’ food security raw scores into one of the three food security categories. Respondents were provided the option to answer “I do not know” or “I prefer not to answer” for each of the six screening questions. If respondents chose one of these options for ≥1 screening question and a minimum score of 2 could be calculated, the FI raw score was categorized as “low food security” (raw score 2–4). If a raw score of <2 or no score was calculated based on these incomplete responses, the respondent’s FI status was categorized as “unable to calculate.”

This study was approved by the institutional review board and was supported through grant funding provided by a 2021 AAP Community Access to Child Health Implementation Grant.

### Statistical Analysis

We collected all data through Qualtrics, supported by Brown University. Data was exported into Excel (Microsoft Corporation, Redmond, WA) for coding, and analysis was performed using SAS version 9.4 (SAS Institute, Inc, Cary, NC). All questions left unanswered in Qualtrics were coded as missing. We performed descriptive statistical analyses and reported the results as frequencies and proportions, with median and interquartile ranges (IQR) calculated. Bivariate analysis was performed to establish associations between respondents’ food security status and various sociodemographic factors, with report of 95% confidence intervals (CI). We performed chi-square tests and Wilcoxon rank-sum tests for comparative analyses, when appropriate.

## RESULTS

### Sociodemographics and Health Statistics

Between February–October 2022, there were 42,697 visits to the PED; the number of patients cared for in rooms/areas where recruitment posters were not available is unknown. A total of 846 visits were associated with initiated surveys (2% of total visits). We excluded 234 responses (27.7% of initiated surveys) from the analysis due to lack of consent or missing responses to all six USDA screening questions, leaving 612 surveys (72.3% of initiated surveys and 1.4% of total visits) for analysis.

Most of the surveys (75.5%) were completed in English by respondents who identified English as their preferred language, 7.8% were completed in English by respondents who identified Spanish as their preferred language, and 15.7% were completed in Spanish by respondents who identified Spanish as their preferred language. Nearly 50% of respondents identified as White and non-Hispanic, respectively, with approximately 80% identifying as female (Table 1). Nearly 90% of respondents were the biological parent of the PED patient.

**Table 1.** Survey respondents’ sociodemographics and food security status, N = 612.

	N (%)^a^
Survey language	
English	516 (84.3)
Spanish	96 (15.7)
Primary language	
English	437 (71.4)
Spanish	136 (22.2)
Other/missing	39 (6.4)
Sex	
Male	94 (15.4)
Female	482 (78.8)
Non-binary	4 (0.7)
Missing/prefer not to answer	32 (5.2)
Race	
Black	51 (8.3)
White	304 (49.7)
Other^b^	189 (30.9)
Missing	68 (11.1)
Ethnicity	
Hispanic	271 (44.3)
Non-Hispanic	294 (48.0)
Prefer not to answer	18 (2.9)
Missing	29 (4.7)
Household annual income	
Under $15,000	120 (19.6)
$15,000–$24,999	63 (10.3)
$25,000–$34,999	132 (21.6)
$35,000–$49,999	65 (10.6)
$50,000–$74,999	69 (11.3)
More than $75,000	100 (16.3)
Unsure/prefer not to answer	51 (8.4)
Missing	12 (2.0)
Housing^c^	
House/apartment owned	188 (30.7)
House/apartment rented	372 (60.8)
Other	24 (3.9)
Temporary/no housing	8 (1.3)
Prefer not to answer	8 (1.3)
Missing	12 (2.0)
Employment status	
Unemployed	185 (30.2)
Full-time	271 (44.3)
Part-time	102 (16.7)
Other/prefer not to answer/missing	54 (8.8)
Insurance type^d^	
Private insurance	312 (51.0)
Government insurance	217 (35.5)
Other	10 (1.6)
None	17 (2.8)
Missing/prefer not to answer/not sure	56 (9.2)

a Percent may not equal 100 due to rounding.

b Other category includes American Indian, Alaska Native, Asian, Native Hawaiian, and “other.”

c Other category includes rented room/boarding house, mobile home/trailer, and “other.”

d Private insurance includes Aetna (N = 8, 1.3%); Blue Cross Blue Shield (N = 98, 16.0%); Blue Chip (N = 67, 10.9%); Tufts (N = 67, 10.7%); United Healthcare (N = 72, 11.8%). Government insurance includes Neighborhood Health Plan, state Medicaid (N = 174, 28.4%); Medicaid plan not otherwise specified (N = 29, 4.7%); Medicare (N = 8, 1.3%); Rite Care (N = 3, 0.5%); TriCare – Military (N = 3, 0.5%). “Other” insurance category is a non-specified insurance (N = 10, 1.6%).

The mean number of household family members for respondents was 4.1 (IQR 3–5, SD 1.4), with 62% living in rented houses or apartments and 31.3% in owned houses (Table 1). Among respondents, 29.9% had annual household incomes <$25,000, with a 2022 federal poverty level (FPL) for a family of 4 of $27,750.^13^ An additional 32.2% had annual household incomes between $25,000–<$50,000, 15.7% between $50,000–<$100,000, 11.9% >$100,000, and 10.3% not reported. Full- or part-time employment was reported by 61% of respondents and over 88% had health insurance (Table 1). Additionally, 271 respondents (44.3%) had ≤ a high school degree or general equivalency diploma, 114 (18.6%) had a business or trade certificate or two-year college degree, and 126 (20.6%) had a four-year college or graduate degree. More than one-third of respondents (226, 36.9%) denied having a partner or spouse in the home. Respondents reported that 83% of the children that they accompanied to the ED had access to pediatric primary care.

### Food Security Status

Food insecurity was demonstrated by 56.7% of the respondents (Table 2), high or marginal food security was demonstrated in 35.3%, and 8% were unable to be categorized based on “I do not know” and “prefer not to answer” responses to the survey questions. Additionally, 13.9%, 65.4%, and 89.2% of respondents in the high, low, and very low food security groups, respectively, reported that their family had to choose between food and other needs, such as paying for housing, utilities, and/or clothing, within the prior 12 months.

**Table 2.** Survey respondents’ food security status.

	N (%)^a^	95% CI of Percentage
USDA food insecurity category (N = 612)		
High or marginal food security	216 (35.3)	31.5, 39.2
Low food security	153 (25.0)	21.6, 28.4
Very low food security	194 (31.7)	28.0, 35.6
Unable to be calculated based on responses	49 (8.0)	6.0, 10.5
Choosing between food and other needs, by food security status^b^ (N = 563)		
High or marginal food security (n = 216)	30 (13.9)	9.6, 19.2
Low food security (n = 153)	100 (65.4)	57.3, 72.9
Very low food security (n = 194)	173 (89.2)	83.9, 93.2

a Percent may not equal 100 due to rounding.

b Respondents were specifically asked if they had to choose between spending money on food or other needs, including rent, utilities, medical care, etc., within the last 12 months. Affirmative responses are reported here.

*CI*, confidence interval; *USDA*, United States Department of Agriculture.

Bivariate analyses revealed statistically significant differences in awareness and utilization of supplemental food services based on respondents’ household food security status, household income, and primary language (Table 3). Overall, respondents reported highest awareness of SNAP compared to WIC, food banks, and free lunch programs. The most used service overall was SNAP. The lowest proportion of awareness and utilization among respondents was for local food banks. Except for SNAP, households with high food security and with household incomes >$50,000 annually had statistically significantly higher proportions of awareness of all supplemental food services, despite the lowest utilization of these services (Table 3). Notably, there was no statistically significant difference in the proportion of respondents with high, low, or very low food security who used free school lunches (*P* = 0.90), nor was there a statistically significant difference in utilization of free school based on primary language (*P* = 0.19). Despite no significant difference in the proportion of respondents with English vs Spanish as their primary language who used most supplemental food services, there were statistically significant differences in awareness of all these services based on primary language (Table 3).

**Table 3.** Awareness^a^ vs utilization of supplemental food services by food security status, annual household income, and primary language.

3a. Food security status					
	High food security (n = 216)	Low food security (n = 153)	Very low food security (n = 194)		*P*-value
Awareness					
WIC	167 (77.3)	87 (57.2)	110 (56.7)		<.001
SNAP	177 (81.9)	123 (80.9)	163 (84.0)		0.67
Food banks	144 (66.7)	65 (42.8)	83 (42.8)		<.001
Free lunch	154 (71.3)	71 (46.7)	83 (42.8)		<.001
Utilization					
WIC	33 (15.3)	43 (28.1)	52 (26.9)		<.001
SNAP	47 (21.8)	57 (37.3)	75 (38.9)		<.001
Food banks	13 (6.0)	20 (13.1)	33 (17.1)		<.001
Free lunch	44 (20.4)	32 (20.9)	43 (22.3)		0.90
3b. Annual household income					
	<$25,000 (n = 183)	$25,000 – <$50,000 (n = 197)	$50,000 – <$100,000 (n = 96)	≥$100,000 (n = 73)	*P*-value
Awareness					
WIC	101 (55.2)	128 (65.3)	69 (71.9)	57 (78.1)	<.01
SNAP	155 (84.7)	161 (82.1)	76 (79.2)	58 (79.5)	0.63
Food banks	70 (38.3)	93 (47.5)	71 (74.0)	54 (74.0)	<.001
Free lunch	78 (42.6)	95 (48.5)	68 (70.8)	58 (79.5)	<.001
Utilization					
WIC	53 (29.1)	58 (29.6)	5 (5.2)	2 (2.7)	<.001
SNAP	109 (59.9)	56 (28.6)	7 (7.3)	2 (2.7)	<.001
Food banks	26 (14.3)	28 (14.3)	10 (10.4)	0 (0)	<.01
Free lunch	40 (22.0)	52 (26.5)	17 (17.7)	7 (9.6)	0.02
3c. Primary language spoken					
	English (n = 437)	Spanish (n = 136)			*P*-value
Awareness					
WIC	307 (70.3)	65 (47.8)			<.001
SNAP	379 (86.7)	94 (69.1)			<.001
Food banks	257 (58.8)	39 (26.7)			<.001
Free lunch	274 (62.7)	42 (30.9)			<.001
Utilization					
WIC	84 (19.2)	48 (35.3)			<.001
SNAP	136 (31.1)	49 (36.0)			0.14
Food banks	47 (10.8)	20 (14.7)			0.43
Free lunch	97 (22.2)	23 (16.9)			0.19

a Respondents were asked if they had “ever heard of” the food services.

*WIC*, Women, Infants & Children Program; *SNAP*, Supplemental Nutrition Assistance Program.

Finally, 148 respondents (24.2%) requested follow-up with a resource advocate for assistance with food resources, including 74 respondents with very low food security (51.4%), 46 with low food security (31.9%), 21 with high food security (14.6%), and seven (4.9%) for whom the security status was unable to be calculated.

## DISCUSSION

The COVID-19 pandemic had tremendous effects on families, with previously reported increases in FI among surveys performed in general populations.^1^ ^–^ ^4^ The anonymous, electronic FI survey containing sociodemographic questions and six validated USDA FI screening questions revealed that 56.7% of respondents surveyed in an urban, tertiary, PED had some degree of household FI. Although baseline FI data among patients seen in the PED is unknown, this prevalence is markedly higher than the proportion of households in the study state with children <18 years of age reporting FI in 2021 (25%) and 2022 (41%).^14^ The prevalence of FI in this population is also notably higher than pre-COVID-19 reports in PEDs,^7^ ^,^ ^8^ as well as higher than reports of general populations collected during the pandemic nationally.^1^ ^–^ ^4^ This study took place two years into the COVID-19 pandemic, after many federal and state legislative changes had occurred that may have impacted food security. It has been suggested that the observed increases in FI during this time was in part due to inflation and discontinuation of some COVID-19 relief programs that mitigated FI.^15^

Despite a state-wide increase in FI among households with children <18 years of age,^14^ an even higher prevalence was seen in the PED setting and is likely multifactorial. First, the ED serves a high-risk population, with increased rates of ED visits among FI families compared to those who are not FI.^6^ ^,^ ^7^ Thus, the patient population is likely to experience an overall higher prevalence of FI. Second, the anonymity of the electronic screening tool may have supported respondents’ willingness to provide accurate information about their family’s food security. While our study’s higher prevalence of FI aligns with reports of increased FI during the COVID-19 pandemic, selection bias may have played a role due to the voluntary nature of our enrollment via posters and electronic surveys. This method could have led to an over-representation of families facing greater food-related hardships and/or those with access to and comfort with using smartphone technology. However, Gayle et al and Gonzalez et al have demonstrated that most caregivers preferred an electronic screening modality over verbal/face-to-face screening for social determinants of health,^7^ ^,^ ^11^ and among those who respond electronically, a higher prevalence of FI was found.^7^

Food insecurity is multifactorial, and household income does not necessarily reflect the financial needs and hardships of families. Although families may have household incomes exceeding the FPL, the high cost of living, utilities, and other expenses may limit the availability of funds for adequate food, resulting in higher rates of FI than rates of poverty. One report notes that one-third of households with FI reported a household income between 100–200% of the FPL, with an additional third reporting over 200% the FPL.^16^ Similarly in this cohort, 169 respondents (27.6) had an annual household income >$50,000, approaching or exceeding 200% of the FPL for a family of four ($55,000 in 2022),^13^ and yet 41(24.2%) in these income categories still were categorized as FI based on their responses. Additionally highlighting the complexities of food security in the setting of other household necessities, nearly 90% of respondents in this survey with very low food security, 65% with low food security, and 14% with high food security reported having had to choose between food and other necessities in the preceding 12 months. Furthermore, nearly 15% of the respondents who requested follow-up from a resource advocate were categorized as having high food security based on survey responses. This could be due to ongoing needs despite currently not meeting the screening threshold for FI, not answering all FI questions truthfully (thus not capturing their accurate food security status), or other reasons.

There were also notable differences in respondents’ awareness and utilization of federal and local supplemental food services when analyzed by FI status, primary language, and household income, demonstrating key gaps within this high-risk population. Despite 83% of respondents reporting that their children had access to pediatric primary care, where the majority of FI screening and intervention generally takes place, less than half of households with low and very low food security reported awareness of local food banks and free school lunches. Approximately 60% had heard of WIC, and 84% had heard of SNAP, with even lower utilization of these services. Similarly, Coleman-Jenson et al previously reported that only 55% of eligible FI households participated in WIC, SNAP, and/or free school-lunch programs.^4^ Some families who do identify primary care physicians may have limited availability to access routine care during regular business hours, thus missing opportunities for screening and intervention, and alternatively seeking care in urgent care or EDs where screening is not the standard of care. Additionally, depending on in-office screening methods (eg, paper, verbal, electronic) and limitations in time allotted for office visits and resource availability, primary care offices may not identify all FI families and/or be able to meet the needs of all its patients. Furthermore, primary care office staff may not be aware of all available local, statewide, and federal resources and eligibility criteria for patients who may qualify. A cycle of poor access, poor screening, inadequate guidance, and negative health outcomes subsequently develops.

The proportion of respondents in the high food security category as well as those with household incomes >$50,000 who reported awareness of all the supplemental food services was significantly higher than the proportion of respondents with low and very low food security and household incomes <$50,000. High resource awareness among food secure families and greater annual household incomes could be due to overall higher education and knowledge of social services even when they are not needed, awareness because of past or current utilization, or other reasons. In this cohort, respondents with the overall highest awareness of supplemental food services had the overall lowest utilization of services when stratified by household income and food security status. Based on these findings, those with the highest knowledge are not necessarily those who are in need. This data suggests that families who are food secure may not necessarily be food secure because of utilization of services; however, additional studies need to be done to further elucidate reasons for these findings.

Another notable finding is that the proportion of respondents who used free school lunches was not statistically significant regardless of food security status. This is likely due to legislation including the Families First Coronavirus Response Act that was put in place during the COVID-19 pandemic to allow access to free school lunches for all children regardless of income.^17^ Unfortunately, despite universal accessibility to free school lunches, approximately 25% or fewer children in households with incomes <$50,000 annually and in the low and very low food security categories, regardless of primary language, participated in this service. Significant stigma around using free school meals can lead to children not participating in the program despite qualifying,^18^ which may play a role in these findings. Considering legislative changes made during the pandemic to provide access to free school meals universally, additional studies are required to understand the impact of these programmatic changes over time.

While it is possible that respondents misunderstood or incorrectly answered questions related to awareness and utilization or did not know of these programs by name, these findings warrant further exploration. Barriers to awareness and utilization are well known, including English-language proficiency, difficulty navigating the complex application processes via phone, online, or in person, and lack of transportation and access to government offices and/or local food resources particularly during regular business hours, among other factors. Families are often not aware of eligibility criteria, particularly if they are not provided this information at healthcare visits, in schools, and through community outreach programs. Although undocumented families may qualify for state and federal assistance services, they may be reluctant to identify themselves to government programs. Although details of immigration status, exact household income, prior utilization of services, the status of pending immigration applications, and barriers to access are unknown among this study cohort, the differences in awareness and utilization are striking, and reiterate the need for further outreach and intervention among the most vulnerable populations.

## LIMITATIONS

There are important limitations to this study to consider. The first is missing data. While over 800 respondents initiated the study, many did not complete the USDA screening questions, thus excluding them from analysis. Among those who did complete the screening questions, not all completed the sociodemographic, awareness, and utilization questions, potentially impacting the results of our analyses. Second is selection bias, which may have been influenced by factors such as respondent interest in the survey, lack of direct recruitment, general and medical literacy, and access to smartphones to complete the survey. Because there was no direct recruitment, respondents with limited literacy and/or those who spoke a language other than English and Spanish also were missed, potentially introducing additional selection bias and limiting generalizability.

The recruitment poster specifically excluded the words “food insecurity” with the intent of trying to recruit respondents from all socioeconomic backgrounds. Because there was a small gift-card incentive offered for participation in the survey, some degree of selection bias likely remained, with those with higher needs primarily responding. However, it is notable that over 16% of respondents reported a household income above $75,000 per year, >250% of the FPL.^13^ Further, approximately 1.4% of the annual PED volume completed surveys, which may not be representative of the general PED population at the study site or in other ED settings, limiting broader generalizability. However, the proportion surveyed is comparable to that of other studies with similar methodology (1.8%^8^ and 0.5%^10^) and demonstrated similar increases in FI reported during the pandemic.^1^ ^,^ ^2^

Other potential biases are important to acknowledge, including the possibility of miscalculation bias, as respondents’ answers may not have been accurate if they did not remember circumstances correctly or if they were not familiar with the names of supplemental food services, among other reasons. There may have been a contribution of social desirability bias, with respondents not accurately reporting their income, awareness, and utilization of services. The ability to take the survey anonymously may have mitigated this bias, however.

Among respondents who provided contact information to receive an incentive gift card, there were no duplicate names or addresses provided. However, a potential limitation includes repeat enrollment at subsequent visits among individuals who did not provide contact information. An additional limitation is that the six questions used are aimed at assessing *household* FI and responses may not directly reflect the food security status of the children living in the homes. Although children may not directly experience household FI, understanding the household dynamics and needs is still critical to identifying disparities and populations in need and identifying opportunities for interventions before children are impacted.

## CONCLUSION

The COVID-19 pandemic had significant impacts on families, with an increase in food insecurity nationally. This study, aligned with prior published research, demonstrates that the ED is an important location for FI screening. Given the high needs of the patient population seen in the ED and this study’s striking finding that more than one in two households screened had some degree of FI, additional studies must be done to optimize FI screening, including determining the best screening modality and interventions for this high-risk population.
